# 4′-(Morpholino­meth­yl)biphenyl-2-carbonitrile

**DOI:** 10.1107/S1600536810025109

**Published:** 2010-07-03

**Authors:** Weiwei SiMa

**Affiliations:** a Ordered Matter Science Research Center, Southeast University, Nanjing 210096, People’s Republic of China

## Abstract

In the title compound, C_18_H_18_N_2_O, the morpholine ring adopts a chair conformation and the dihedral angle between the aromatic rings is 49.16 (7)°. In the crystal, weak C—H⋯π inter­actions may help to establish the packing.

## Related literature

For background to ligands related to the title compound, see: Li *et al.* (2008 ▶); Zhang *et al.* (2009 ▶).
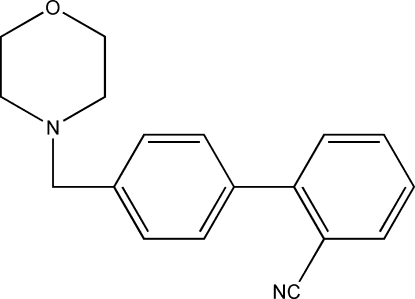


## Experimental

### 

#### Crystal data

C_18_H_18_N_2_O
*M* *_r_* = 278.34
Monoclinic, 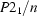

*a* = 10.924 (6) Å
*b* = 10.891 (5) Å
*c* = 12.943 (7) Å
β = 93.269 (7)°
*V* = 1537.4 (13) Å^3^
*Z* = 4
Mo *K*α radiation
μ = 0.08 mm^−1^
*T* = 293 K
0.20 × 0.20 × 0.20 mm

#### Data collection

Rigaku SCXmini diffractometer
Absorption correction: multi-scan (*CrystalClear*; Rigaku, 2005 ▶) *T*_min_ = 0.985, *T*_max_ = 0.985
16364 measured reflections
3493 independent reflections
2757 reflections with *I* > 2σ(*I*)
*R*_int_ = 0.042

#### Refinement

*R*[*F* ^2^ > 2σ(*F* ^2^)] = 0.053
*wR*(*F* ^2^) = 0.138
*S* = 1.10
3493 reflections
190 parameters
H-atom parameters constrained
Δρ_max_ = 0.14 e Å^−3^
Δρ_min_ = −0.15 e Å^−3^

### 

Data collection: *CrystalClear* (Rigaku, 2005 ▶); cell refinement: *CrystalClear*; data reduction: *CrystalClear*; program(s) used to solve structure: *SHELXS97* (Sheldrick, 2008 ▶); program(s) used to refine structure: *SHELXL97* (Sheldrick, 2008 ▶); molecular graphics: *SHELXTL* (Sheldrick, 2008 ▶); software used to prepare material for publication: *SHELXL97*.

## Supplementary Material

Crystal structure: contains datablocks I, global. DOI: 10.1107/S1600536810025109/hb5523sup1.cif

Structure factors: contains datablocks I. DOI: 10.1107/S1600536810025109/hb5523Isup2.hkl

Additional supplementary materials:  crystallographic information; 3D view; checkCIF report

## Figures and Tables

**Table 1:** Hydrogen-bond geometry (Å, °) *Cg*2 is the centroid of the C6–C11 ring.

*D*—H⋯*A*	*D*—H	H⋯*A*	*D*⋯*A*	*D*—H⋯*A*
C1—H1*A*⋯*Cg*2^i^	0.96	2.76	3.638 (3)	151
C16—H16*A*⋯*Cg*2^ii^	0.93	2.87	3.746 (3)	157

Symmetry codes: (i) 
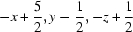
; (ii) 
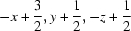
.

**Fig. 1.**
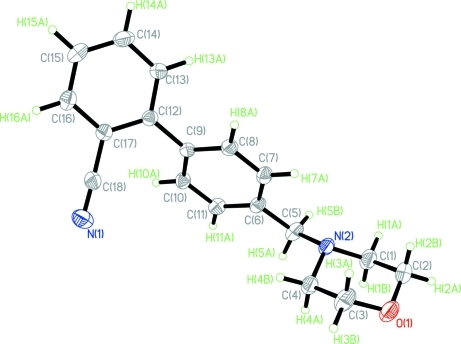
The molecular structure of (I) with displacement ellipsoids drawn at the 30% probability level, and all H atoms have been omitted for clarity.

**Fig. 2.**
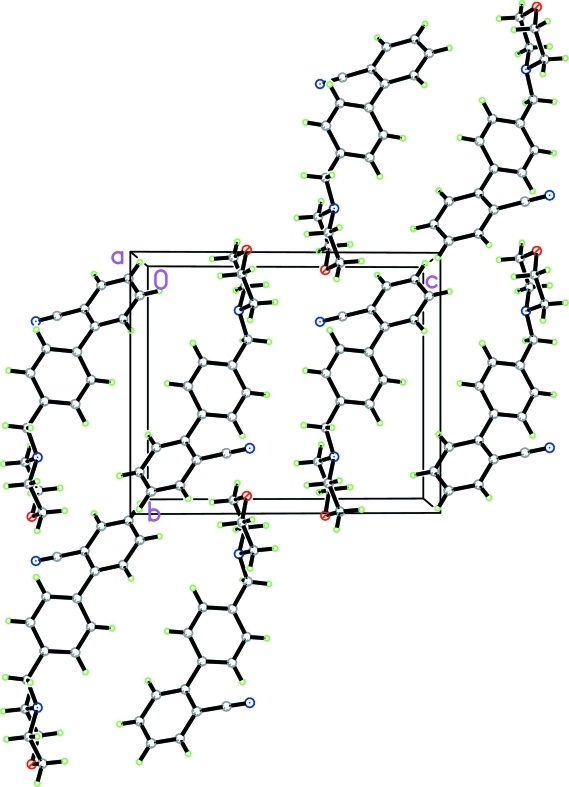
A view of the packing of the title compound, stacking along the a axis. Dashed lines indicate hydrogen bonds.

## supplementary crystallographic information

### Experimental

A solution of 4'-bromomethylbiphenyl-2-carbonitrile (10 mmol) in acetone was added dropwise to a mixture of morpholine (10 mmol) and potassium carbonate anhydrous (10 mmol) at 331 K with stirring for 6 h; the reaction solution was then filted. Colourless prisms of (I) were formed after several weeks by slow evaporation of the solvent at room temperature. The compound shows no dielectric irregularity in the temperature range of 93–352 K.

### Refinement

Positional parameters of all the H atoms were calculated geometrically and were allowed to ride on the C, N atoms to which they are bonded, with C—H =0.93 to 0.97 Å, *U*_iso_(H) = 1.2 *U*_eq_(C), N—H = 0.89 Å, *U*_iso_(H)= 1.5 *U*_eq_(N).

### Crystal data

**Table:**

C_18_H_18_N_2_O	*F*(000) = 592
*M**_r_* = 278.34	*D*_x_ = 1.203 Mg m^−^^3^
Monoclinic, *P*2_1_/*n*	Mo *K*α radiation, λ = 0.71073 Å
Hall symbol: -P 2yn	Cell parameters from 3548 reflections
*a* = 10.924 (6) Å	θ = 2.4–27.4°
*b* = 10.891 (5) Å	µ = 0.08 mm^−^^1^
*c* = 12.943 (7) Å	*T* = 293 K
β = 93.269 (7)°	Prism, colourless
*V* = 1537.4 (13) Å^3^	0.20 × 0.20 × 0.20 mm
*Z* = 4	

### Data collection

**Table:**

Rigaku SCXmini diffractometer	3493 independent reflections
Radiation source: fine-focus sealed tube	2757 reflections with *I* > 2σ(*I*)
graphite	*R*_int_ = 0.042
Detector resolution: 13.6612 pixels mm^-1^	θ_max_ = 27.5°, θ_min_ = 2.4°
ω scans	*h* = −14→14
Absorption correction: multi-scan (*CrystalClear*; Rigaku, 2005)	*k* = −14→14
*T*_min_ = 0.985, *T*_max_ = 0.985	*l* = −16→16
16364 measured reflections	

### Refinement

**Table:**

Refinement on *F*^2^	Primary atom site location: structure-invariant direct methods
Least-squares matrix: full	Secondary atom site location: difference Fourier map
*R*[*F*^2^ > 2σ(*F*^2^)] = 0.053	Hydrogen site location: inferred from neighbouring sites
*wR*(*F*^2^) = 0.138	H-atom parameters constrained
*S* = 1.10	*w* = 1/[σ^2^(*F*_o_^2^) + (0.0609*P*)^2^ + 0.152*P*] where *P* = (*F*_o_^2^ + 2*F*_c_^2^)/3
3493 reflections	(Δ/σ)_max_ < 0.001
190 parameters	Δρ_max_ = 0.14 e Å^−^^3^
0 restraints	Δρ_min_ = −0.14 e Å^−^^3^

### Special details

**Table:**

Geometry. All e.s.d.'s (except the e.s.d. in the dihedral angle between two l.s. planes) are estimated using the full covariance matrix. The cell e.s.d.'s are taken into account individually in the estimation of e.s.d.'s in distances, angles and torsion angles; correlations between e.s.d.'s in cell parameters are only used when they are defined by crystal symmetry. An approximate (isotropic) treatment of cell e.s.d.'s is used for estimating e.s.d.'s involving l.s. planes.
Refinement. Refinement of *F*^2^ against ALL reflections. The weighted *R*-factor *wR* and goodness of fit *S* are based on *F*^2^, conventional *R*-factors *R* are based on *F*, with *F* set to zero for negative *F*^2^. The threshold expression of *F*^2^ > σ(*F*^2^) is used only for calculating *R*-factors(gt) *etc*. and is not relevant to the choice of reflections for refinement. *R*-factors based on *F*^2^ are statistically about twice as large as those based on *F*, and *R*- factors based on ALL data will be even larger.

### Fractional atomic coordinates and isotropic or equivalent isotropic displacement parameters (Å^2^)

**Table:**

	*x*	*y*	*z*	*U*_iso_*/*U*_eq_	
C5	1.14259 (16)	0.82069 (14)	0.13367 (14)	0.0528 (4)	
H5A	1.1325	0.8304	0.0600	0.063*	
H5B	1.2288	0.8226	0.1527	0.063*	
C9	0.97335 (13)	1.12626 (12)	0.28571 (10)	0.0365 (3)	
N2	1.09293 (12)	0.70241 (11)	0.16311 (10)	0.0448 (3)	
C12	0.92172 (13)	1.23165 (12)	0.34199 (11)	0.0380 (3)	
C17	0.82704 (13)	1.30573 (13)	0.29907 (11)	0.0395 (3)	
C7	1.04111 (15)	0.91490 (13)	0.28539 (12)	0.0459 (4)	
H7A	1.0501	0.8374	0.3203	0.055*	
C10	1.01117 (14)	1.13799 (13)	0.18511 (11)	0.0420 (3)	
H10A	1.0006	1.2150	0.1497	0.050*	
C6	1.08033 (14)	0.92683 (13)	0.18531 (12)	0.0421 (4)	
O1	1.00038 (13)	0.45991 (10)	0.13465 (10)	0.0654 (4)	
C16	0.78280 (14)	1.40534 (14)	0.35368 (13)	0.0484 (4)	
H16A	0.7205	1.4542	0.3238	0.058*	
C18	0.76939 (14)	1.27795 (14)	0.19868 (13)	0.0471 (4)	
C11	1.06370 (14)	1.03952 (14)	0.13615 (12)	0.0452 (4)	
H11A	1.0885	1.0491	0.0666	0.054*	
C8	0.98879 (15)	1.01272 (13)	0.33456 (12)	0.0439 (4)	
H8A	0.9631	1.0025	0.4038	0.053*	
C13	0.96826 (16)	1.26019 (14)	0.44174 (12)	0.0493 (4)	
H13A	1.0335	1.2114	0.4731	0.059*	
C4	0.97391 (15)	0.67913 (14)	0.11041 (14)	0.0505 (4)	
H4A	0.9831	0.6723	0.0373	0.061*	
H4B	0.9195	0.7463	0.1219	0.061*	
C14	0.92365 (18)	1.35790 (16)	0.49598 (13)	0.0576 (5)	
H14A	0.9561	1.3749	0.5650	0.069*	
C15	0.83137 (17)	1.43118 (15)	0.45173 (14)	0.0556 (4)	
H15A	0.8015	1.4998	0.4894	0.067*	
N1	0.71962 (15)	1.25687 (16)	0.12030 (12)	0.0694 (5)	
C1	1.17405 (17)	0.59987 (15)	0.14313 (16)	0.0639 (5)	
H1A	1.2532	0.6137	0.1773	0.077*	
H1B	1.1843	0.5923	0.0702	0.077*	
C3	0.92168 (18)	0.56140 (15)	0.14975 (17)	0.0666 (5)	
H3A	0.9090	0.5703	0.2222	0.080*	
H3B	0.8435	0.5460	0.1144	0.080*	
C2	1.1182 (2)	0.48294 (16)	0.18227 (17)	0.0732 (6)	
H2A	1.1716	0.4151	0.1701	0.088*	
H2B	1.1113	0.4901	0.2556	0.088*	

### Atomic displacement parameters (Å^2^)

**Table:**

	*U*^11^	*U*^22^	*U*^33^	*U*^12^	*U*^13^	*U*^23^
C5	0.0498 (10)	0.0402 (8)	0.0696 (11)	−0.0040 (7)	0.0130 (8)	−0.0107 (7)
C9	0.0376 (8)	0.0340 (7)	0.0373 (7)	−0.0013 (6)	−0.0025 (6)	−0.0027 (6)
N2	0.0462 (7)	0.0338 (6)	0.0542 (8)	0.0031 (5)	0.0015 (6)	−0.0064 (5)
C12	0.0419 (8)	0.0338 (7)	0.0384 (7)	−0.0034 (6)	0.0017 (6)	−0.0004 (6)
C17	0.0372 (8)	0.0379 (8)	0.0434 (8)	−0.0038 (6)	0.0033 (6)	−0.0002 (6)
C7	0.0559 (10)	0.0331 (8)	0.0487 (9)	−0.0002 (6)	0.0027 (7)	0.0025 (6)
C10	0.0462 (9)	0.0363 (7)	0.0437 (8)	0.0003 (6)	0.0034 (6)	0.0035 (6)
C6	0.0402 (8)	0.0353 (8)	0.0511 (9)	−0.0045 (6)	0.0041 (6)	−0.0060 (6)
O1	0.0846 (10)	0.0374 (6)	0.0742 (9)	−0.0038 (6)	0.0041 (7)	−0.0066 (5)
C16	0.0425 (9)	0.0417 (8)	0.0617 (10)	0.0020 (6)	0.0078 (7)	−0.0027 (7)
C18	0.0405 (9)	0.0480 (9)	0.0525 (9)	0.0043 (7)	−0.0011 (7)	−0.0003 (7)
C11	0.0488 (9)	0.0445 (9)	0.0431 (8)	−0.0032 (7)	0.0101 (7)	0.0000 (6)
C8	0.0540 (9)	0.0395 (8)	0.0381 (8)	−0.0013 (7)	0.0018 (6)	0.0007 (6)
C13	0.0590 (10)	0.0444 (9)	0.0433 (8)	0.0013 (7)	−0.0068 (7)	−0.0042 (7)
C4	0.0464 (9)	0.0406 (8)	0.0644 (10)	0.0029 (7)	0.0015 (7)	−0.0049 (7)
C14	0.0777 (13)	0.0520 (10)	0.0428 (9)	−0.0052 (9)	0.0003 (8)	−0.0113 (7)
C15	0.0628 (11)	0.0451 (9)	0.0603 (11)	−0.0028 (8)	0.0164 (8)	−0.0150 (8)
N1	0.0603 (10)	0.0852 (12)	0.0608 (10)	0.0075 (8)	−0.0139 (8)	−0.0089 (8)
C1	0.0558 (11)	0.0444 (10)	0.0903 (14)	0.0115 (8)	−0.0053 (10)	−0.0108 (9)
C3	0.0625 (12)	0.0464 (10)	0.0923 (15)	−0.0088 (8)	0.0161 (10)	−0.0087 (9)
C2	0.0919 (16)	0.0431 (10)	0.0824 (14)	0.0137 (10)	−0.0138 (12)	−0.0018 (9)

### Geometric parameters (Å, °)

**Table:**

C5—N2	1.457 (2)	C16—C15	1.376 (2)
C5—C6	1.516 (2)	C16—H16A	0.9300
C5—H5A	0.9600	C18—N1	1.146 (2)
C5—H5B	0.9601	C11—H11A	0.9601
C9—C10	1.394 (2)	C8—H8A	0.9599
C9—C8	1.395 (2)	C13—C14	1.379 (2)
C9—C12	1.4877 (19)	C13—H13A	0.9600
N2—C4	1.456 (2)	C4—C3	1.504 (2)
N2—C1	1.458 (2)	C4—H4A	0.9600
C12—C13	1.395 (2)	C4—H4B	0.9600
C12—C17	1.401 (2)	C14—C15	1.384 (3)
C17—C16	1.396 (2)	C14—H14A	0.9600
C17—C18	1.443 (2)	C15—H15A	0.9600
C7—C8	1.381 (2)	C1—C2	1.512 (3)
C7—C6	1.393 (2)	C1—H1A	0.9600
C7—H7A	0.9601	C1—H1B	0.9599
C10—C11	1.387 (2)	C3—H3A	0.9601
C10—H10A	0.9600	C3—H3B	0.9599
C6—C11	1.390 (2)	C2—H2A	0.9600
O1—C2	1.417 (3)	C2—H2B	0.9599
O1—C3	1.421 (2)		
			
N2—C5—C6	112.08 (13)	C7—C8—H8A	119.4
N2—C5—H5A	109.5	C9—C8—H8A	119.4
C6—C5—H5A	109.0	C14—C13—C12	121.48 (15)
N2—C5—H5B	109.0	C14—C13—H13A	119.2
C6—C5—H5B	109.2	C12—C13—H13A	119.3
H5A—C5—H5B	108.0	N2—C4—C3	109.62 (14)
C10—C9—C8	118.04 (13)	N2—C4—H4A	109.6
C10—C9—C12	121.83 (12)	C3—C4—H4A	109.3
C8—C9—C12	120.10 (13)	N2—C4—H4B	109.8
C4—N2—C5	111.55 (13)	C3—C4—H4B	110.3
C4—N2—C1	108.61 (12)	H4A—C4—H4B	108.2
C5—N2—C1	113.06 (14)	C13—C14—C15	120.30 (15)
C13—C12—C17	117.41 (13)	C13—C14—H14A	120.0
C13—C12—C9	119.78 (13)	C15—C14—H14A	119.7
C17—C12—C9	122.81 (13)	C16—C15—C14	119.81 (15)
C16—C17—C12	121.04 (14)	C16—C15—H15A	120.2
C16—C17—C18	118.22 (14)	C14—C15—H15A	120.0
C12—C17—C18	120.70 (13)	N2—C1—C2	108.95 (16)
C8—C7—C6	120.91 (14)	N2—C1—H1A	109.8
C8—C7—H7A	119.7	C2—C1—H1A	110.3
C6—C7—H7A	119.4	N2—C1—H1B	110.3
C11—C10—C9	120.56 (13)	C2—C1—H1B	109.2
C11—C10—H10A	120.0	H1A—C1—H1B	108.3
C9—C10—H10A	119.5	O1—C3—C4	111.87 (15)
C11—C6—C7	117.96 (14)	O1—C3—H3A	109.6
C11—C6—C5	121.46 (14)	C4—C3—H3A	108.8
C7—C6—C5	120.55 (14)	O1—C3—H3B	109.1
C2—O1—C3	110.13 (13)	C4—C3—H3B	109.4
C15—C16—C17	119.96 (15)	H3A—C3—H3B	108.0
C15—C16—H16A	120.0	O1—C2—C1	112.15 (15)
C17—C16—H16A	120.0	O1—C2—H2A	109.7
N1—C18—C17	177.55 (18)	C1—C2—H2A	109.5
C10—C11—C6	121.37 (14)	O1—C2—H2B	108.9
C10—C11—H11A	119.4	C1—C2—H2B	108.6
C6—C11—H11A	119.3	H2A—C2—H2B	107.9
C7—C8—C9	121.16 (14)		
			
C6—C5—N2—C4	75.91 (17)	C9—C10—C11—C6	0.0 (2)
C6—C5—N2—C1	−161.33 (14)	C7—C6—C11—C10	0.8 (2)
C10—C9—C12—C13	−129.59 (16)	C5—C6—C11—C10	−177.38 (14)
C8—C9—C12—C13	48.3 (2)	C6—C7—C8—C9	0.1 (2)
C10—C9—C12—C17	50.1 (2)	C10—C9—C8—C7	0.6 (2)
C8—C9—C12—C17	−131.94 (15)	C12—C9—C8—C7	−177.35 (13)
C13—C12—C17—C16	1.0 (2)	C17—C12—C13—C14	−0.4 (2)
C9—C12—C17—C16	−178.70 (13)	C9—C12—C13—C14	179.34 (15)
C13—C12—C17—C18	−176.48 (14)	C5—N2—C4—C3	−175.30 (13)
C9—C12—C17—C18	3.8 (2)	C1—N2—C4—C3	59.43 (18)
C8—C9—C10—C11	−0.7 (2)	C12—C13—C14—C15	−0.5 (3)
C12—C9—C10—C11	177.28 (13)	C17—C16—C15—C14	−0.3 (2)
C8—C7—C6—C11	−0.8 (2)	C13—C14—C15—C16	0.9 (3)
C8—C7—C6—C5	177.36 (14)	C4—N2—C1—C2	−59.12 (19)
N2—C5—C6—C11	−147.09 (15)	C5—N2—C1—C2	176.50 (14)
N2—C5—C6—C7	34.8 (2)	C2—O1—C3—C4	56.2 (2)
C12—C17—C16—C15	−0.7 (2)	N2—C4—C3—O1	−58.5 (2)
C18—C17—C16—C15	176.86 (15)	C3—O1—C2—C1	−56.5 (2)
C16—C17—C18—N1	−39 (4)	N2—C1—C2—O1	58.6 (2)
C12—C17—C18—N1	138 (4)		

### Hydrogen-bond geometry (Å, °)

**Table:**

Cg2 is the centroid of the C6–C11 ring.

**Table:**

*D*—H···*A*	*D*—H	H···*A*	*D*···*A*	*D*—H···*A*
C1—H1A···Cg2^i^	0.96	2.76	3.638 (3)	151
C16—H16A···Cg2^ii^	0.93	2.87	3.746 (3)	157

Symmetry codes: (i) −*x*+5/2, *y*−1/2, −*z*+1/2; (ii) −*x*+3/2, *y*+1/2, −*z*+1/2.
